# Combination of MiR-103a-3p and Mesothelin Improves the Biomarker Performance of Malignant Mesothelioma Diagnosis

**DOI:** 10.1371/journal.pone.0114483

**Published:** 2014-12-03

**Authors:** Daniel G. Weber, Swaantje Casjens, Georg Johnen, Oleksandr Bryk, Irina Raiko, Beate Pesch, Jens Kollmeier, Torsten T. Bauer, Thomas Brüning

**Affiliations:** 1 Center of Molecular Medicine, Institute of Prevention and Occupational Medicine of the German Social Accident Insurance - Institute of the Ruhr-Universität Bochum (IPA), Bochum, Germany; 2 HELIOS Clinic Emil von Behring, Respiratory Disease Clinic Heckeshorn, Berlin, Germany; University of Central Florida, United States of America

## Abstract

**Background:**

For the detection of malignant mesothelioma no single biomarker with reasonable sensitivity and specificity has been described so far. Mesothelin, the most prominent blood-based biomarker, is characterized by high specificity but low sensitivity. It might be reasonable to combine biomarkers of different molecular classes in order to improve the overall performance. The aim of this study was to assess the performance of the combination of mesothelin and miR-103a-3p as blood-based biomarker for mesothelioma.

**Methods/Principal Findings:**

Mesothelin concentration in plasma and miR-103a-3p levels in the cellular blood fraction were analyzed in 43 male mesothelioma patients and 52 male controls formerly exposed to asbestos. For the discrimination of epithelioid and biphasic mesothelioma from asbestos-exposed controls mesothelin and miR-103a-3p showed 74% and 89% sensitivity and 85% and 63% specificity, respectively. For the combination of mesothelin and miR-103a-3p a sensitivity of 95% and a specificity of 81% were calculated.

**Conclusions/Significance:**

The results of this study show that the combination of mesothelin and miR-103a-3p improves the diagnostic performance of individual blood-based biomarker to detect malignant mesothelioma. The obtained results indicate that the use of biomarkers of different molecular classes might be a reasonable approach to assemble a biomarker panel.

## Introduction

Malignant mesothelioma is an aggressive cancer of the serous membranes with increasing incidence worldwide [1]. For the United States 85,000 cases are expected until 2054 [2] whereas for the United Kingdom [3] and Japan [4] 65,000 and 66,000 cases are estimated until 2050. Mesothelioma shows a latency period up to 40 years and median survival is approximately 9–13 months (depending on treatment) from diagnosis [5], because symptoms commonly occur only at late stages of the disease. Thus, the diagnosis of mesothelioma at early stages might be a promising opportunity to improve therapy.

For the detection of cancer at early stages blood-based biomarkers are, in principal, a feasible approach, either for direct diagnosis of the disease or in order to guide suspicious cases to more costly diagnostic methods like High Resolution Computed Tomography (HRCT). Screening for mesothelioma might be clinically important in at-risk collectives composed of subjects formerly exposed to asbestos [6] because occupational exposure to asbestos is the main risk factor for the development of mesothelioma [7]. Proper biomarkers for diagnosis need to fulfill several key features for the application in screening or clinical routine [8]. The four most important features are: (i) minimally-invasive to measure the biomarkers in easily accessible body fluids, (ii) high specificity to avoid false-positive tests in cancer-free subjects, (iii) sufficient sensitivity to detect individuals with cancer, and (iv) robustness against influencing factors.

In recent years, two molecular classes were in focus as blood-based biomarkers for malignant mesothelioma, proteins and microRNAs (miRNAs). Several candidate biomarkers of both molecular classes were described, e.g., mesothelin [9], calretinin [10], fibulin-3 [11], miR-126 [12], miR-625-3p [13], and miR-103a-3p (previous miRBase ID: miR-103) [14].

Up to date, the most prominent biomarker for mesothelioma is mesothelin. However, mesothelin as an individual biomarker is characterized by a high specificity of 89% (95% CI 86; 91%) but a relatively low sensitivity of 58% (95% CI 54; 62%) for the discrimination of mesothelioma patients and asbestos exposed subjects as recently shown in a meta-analysis [15]. Thus, combination of mesothelin with other biomarkers might improve the diagnostic performance.

The aim of this study was to assess the combination of the protein marker mesothelin in plasma and the miRNA miR-103a-3p in the cellular blood fraction in order to enhance the biomarker performance to diagnose malignant mesothelioma.

## Methods

### Ethics statement

All participants of the study provided written informed consent. The study was designed according to rules guarding patient privacy and with the approval from the ethics committee of the Ruhr-Universität Bochum (reference number 3217–08).

### Study population

The study group consisted of 43 male patients with diagnosed mesothelioma including 28 epithelioid (65%), six biphasic (14%) and five sarcomatoid (12%) mesothelioma. The histological subtype was not specified in four cases (9%). None of the patients were treated by surgery, chemotherapy, or radiation therapy before blood collection. The control group consisted of 52 male subjects formerly exposed to asbestos. Detailed characteristics of the study groups are listed in Table 1. Participants were recruited at the HELIOS Clinic Emil von Behring, Berlin, Germany and in participating medical practices of the MoMar study. The MoMar study is a prospective study comprising an annual examination and peripheral blood collection of more than 2.000 German workers formerly exposed to asbestos over a period of up to nine years to identify and validate molecular biomarkers. Twenty-three mesothelioma patients and 17 asbestos-exposed controls were part of a previous screening group identifying miR-103a-3p as a potential biomarker for mesothelioma [14].

**Table 1:** Characteristics of the study groups.

		Mesothelioma cases (N)	Asbestos-exposed controls (N)
Gender	Male	43	52
Age (years)	Median	72	73
	Range	35–85	43–85
Smoking status*	Ever	21	34
	Never	20	18
Histological subtype	Epithelioid	28	
	Biphasic	6	
	Sarcomatoid	5	
	Not specified	4	
Hypertension		20	30
Diabetes mellitus		9	7

*Smoking status is missing for two subjects.

### Blood collection

From each participant peripheral blood was collected in 9.0 ml S-Monovette EDTA gel tubes (Sarstedt, Nümbrecht, Germany) and centrifuged at 2,000×g for ten minutes at room temperature within 30 minutes after blood collection. Plasma was separated from the cellular fraction and both matrices were frozen immediately.

### Measurement of mesothelin

Plasma mesothelin was measured using the ELISA kit MESOMARK (Fujirebio Diagnostics, Inc., Malvern, PA, USA) as described previously [16]. Mesothelin values are presented in Table S1.

### RNA isolation

RNA isolation from 0.5 ml of the cellular fraction was performed using the RiboPure-Blood Kit according to the Alternate Protocol: Isolation of Small RNAs (Life Technologies, Darmstadt, Germany). Concentration of RNA was quantified by measuring the absorbance at 260 nm using a NanoDrop ND-100 spectrophotometer (Thermo Scientific, Waltham, MA, USA).

### Quantitative real-time PCR (qPCR)

TaqMan miRNA Assays (Life Technologies) were used for quantitative miRNA expression analysis of miR-103a-3p as biomarker and miR-125a as reference. In an earlier study of our group using oligonucleotide microarrays to analyze 328 miRNAs, miR-103a-3p was identified as significantly deregulated in mesothelioma patients *vs*. controls formerly exposed to asbestos and *vs.* healthy volunteers of the general population, whereas miR-125a was the most stable miRNA in the analyzed study groups [14]. Quantitative real-time PCR (qPCR) was performed using a 7300 Real-Time PCR System (Life Technologies) as described previously [17], [18]. In brief, 10 ng RNA for reverse transcription reaction and 5 µl cDNA for PCR reaction were used as templates. Samples were analyzed in duplicate and non-template controls were included in all assays. Data analysis was performed as described previously [14] and 2^-dCt^ expression values were used for statistical analysis. Raw Ct values are presented in Table S1.

### Statistical analysis

Statistical analyses were performed using SAS/STAT and SAS/IML software, version 9.3 (SAS Institute Inc., Cary, NC, USA). Box plots with median and inter-quartile range (IQR) were used to depict the distribution of mesothelin and miR-103a-3p. Groups were compared using the non-parametric Wilcoxon rank-sum test. Potential factors influencing the biomarkers were evaluated using multiple linear regression models with log-transformed biomarker values. Effect estimates were given as exp(β) with 95% confidence intervals (CI) and p values. Values of exp(β)>1 and <1 indicate a positive and a negative association between analyzed factor and mesothelin or miRNA-103a-3p, respectively. *PROC LOGISTIC* in SAS was used to determine sensitivities and specificities of mesothelin and miRNA-103a-3p from receiver operating characteristic (ROC) curves illustrating the performance of both biomarkers and their combination to discriminate the studied groups. The ROC curves of the biomarker combination were calculated with mesothelin and miRNA-103a-3 as independent variables in a multiple logistic regression model. The bootstrap procedure (1,000 runs) was used for internal validation of the estimates in the ROC analyses. Biomarker cut-offs were determined with maximum Youden's index (YI) or a false-positive rate (FPR) of 4%. Logistic regression was performed to calculate odds ratios (OR) of a false-positive test in controls and of a false-negative test in mesothelioma cases based on potential influencing factors.

## Results

### Distribution of mesothelin and miRNA-103a-3p

**Figure 1:**
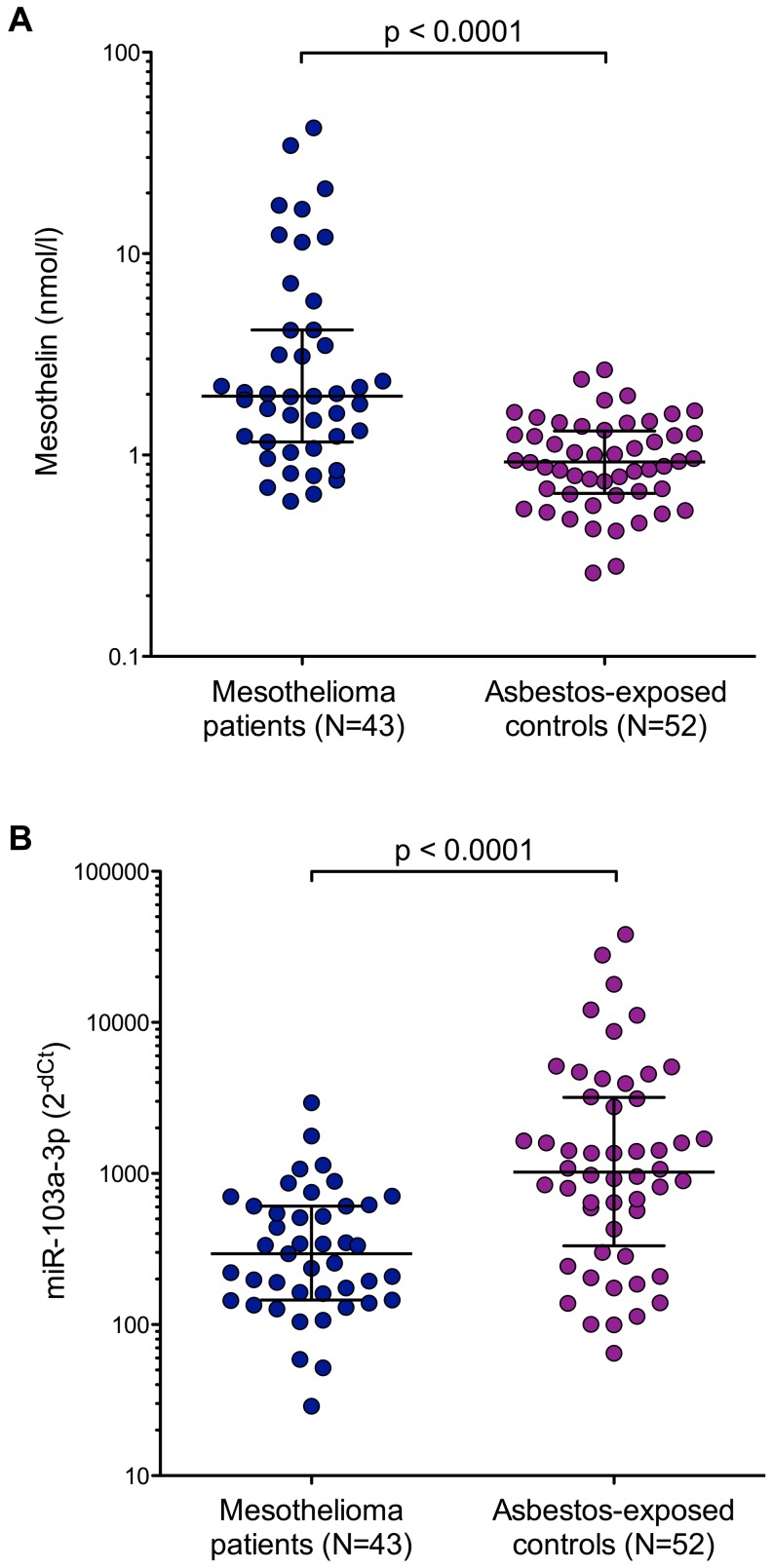
Box plots of mesothelin (A) and miR-103a-3p (B) in mesothelioma patients and asbestos-exposed controls (A). Non-parametric Wilcoxon rank-sum tests were performed to examine group differences. Horizontal bars represent median and inter-quartile range.

Distribution of mesothelin concentrations and normalized miR-103a-3p levels in the two study groups are shown in Figure 1. Median mesothelin concentration for mesothelioma cases was 1.96 nmol/l (IQR 1.16–4.18 nmol/l) whereas for controls median concentration was 0.93 nmol/l (IQR 0.65–1.31 nmol/l), (Figure 1A). For miR-103a-3p mesothelioma cases showed a median level of 294.3 (IQR 145.2–609.2) whereas for controls the median value was 1020.7 (IQR 364.6–3172.9), (Figure 1B). Differences between mesothelioma patients and asbestos-exposed controls were statistically significant for both biomarkers (p<0.001).

**Figure 2:**
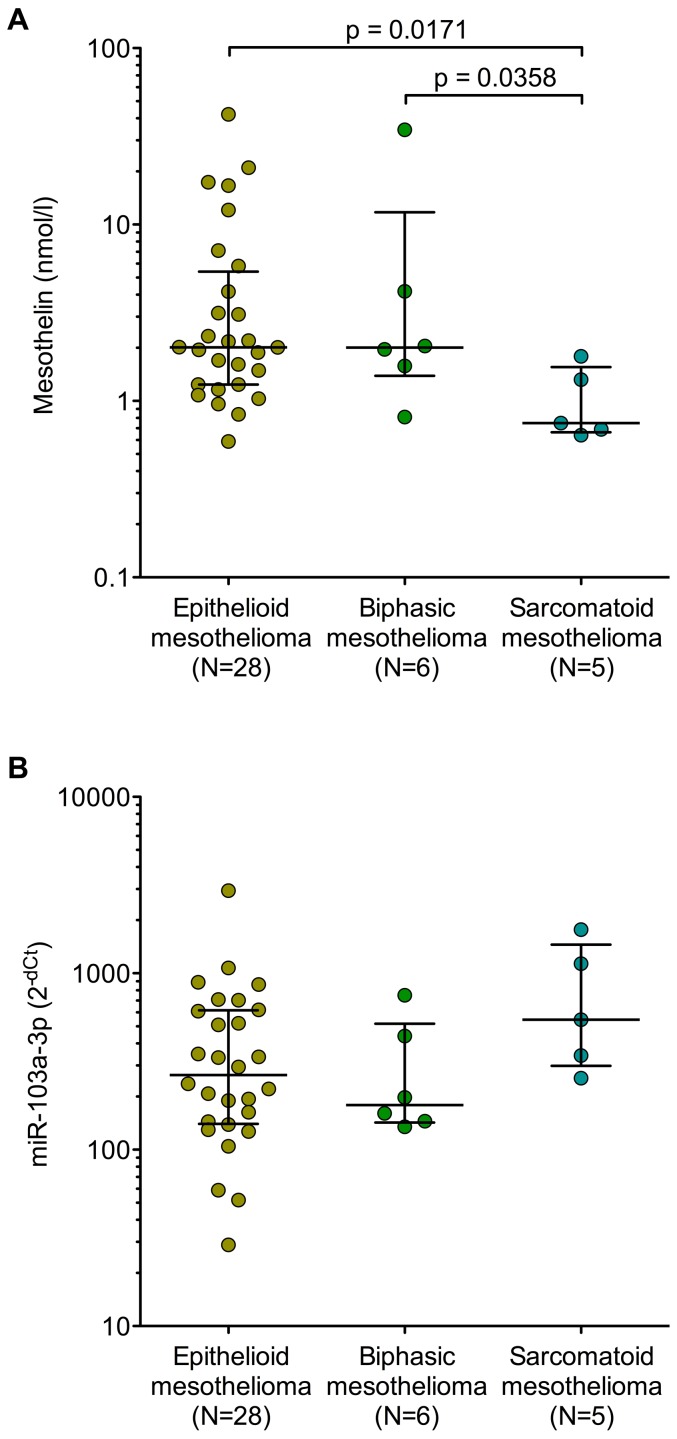
Box plot of mesothelin (A) and miR-103a-3p (B) in histological subtypes of mesothelioma. Non-parametric Wilcoxon rank-sum tests were performed to examine group differences. Horizontal bars represent median and inter-quartile range.

Mesothelin showed a median concentration of 2.01 nmol/l (IQR 1.24–4.99 nmol/l) for epithelioid, 2.01 nmol/l (IQR 1.58–4.18 nmol/l) for biphasic, and 0.75 nmol/l (IQR 0.69–1.32 nmol/l) for sarcomatoid mesothelioma (Figure 2A). Differences were observed for sarcomatoid *vs.* epithelioid (p = 0.017) and sarcomatoid *vs.* biphasic mesothelioma (p = 0.036). Normalized miR-103a-3p levels showed a median value of 265.2 (IQR 141.6–614.9) for epithelioid, 179.0 (IQR 145.2–440.2) for biphasic, and 545.8 (IQR 342.2–1133.5) for sarcomatoid mesothelioma (Figure 2B). Differences between histological subtypes were not significant.

### Study group characteristics

The impact of study group characteristics as potential influencing factors on mesothelin and miRNA-103a-3p are shown in Table 2. The results revealed for the two biomarkers were very similar: both, mesothelin and miR-103a-3p were strongly affected by epithelioid (p<0.001) and biphasic (p<0.001) mesothelioma. Sarcomatoid mesothelioma, age, smoking status, hypertension, and diabetes mellitus did not influence mesothelin and miR-103a-3p. Applying the calculated cut-off based on ROC analysis with maximum YI of the combination of mesothelin and miR-103a-3p (Table 3), sarcomatoid mesothelioma showed an increased OR of 72 (95% CI 5; 982) to be tested false-negative, whereas all other group characteristics showed no significant altered OR to be tested false-negative or false-positive (data not shown).

**Table 2:** Study group characteristics influencing mesothelin and miR-103a-3p.

		Mesothelin			miR-103a-3p		
		Exp(ß)	95% CI	p value	Exp(ß)	95% CI	p value
Intercept		0.37	0.11; 1.25		164.83	23.04; 1179.43	
Mesothelioma (Reference: Controls)	Epithelioid	3.06	2.06; 4.55	<0.001	0.29	0.16; 0.55	<0.001
	Biphasic	3.00	1.49; 6.03	0.002	0.22	0.07; 0.67	0.008
	Sarcomatoid	1.07	0.50; 2.31	0.852	0.61	0.18; 2.07	0.420
	Not specified	4.96	2.17; 11.36	<0.001	0.22	0.06; 0.85	0.028
Age	(10 years)	1.13	0.96; 1.34	0.142	1.24	0.95; 1.62	0.107
Smoking status (Reference: Never)	Ever	1.22	0.85; 1.74	0.279	1.15	0.65; 2.04	0.623
Hypertension (Reference: No)	Yes	0.82	0.58; 1.17	0.271	1.67	0.96; 2.92	0.071
Diabetes mellitus (Reference: No)	Yes	1.20	0.76; 1.89	0.472	0.94	0.45; 1.94	0.859
Adjusted R^2^		0.33			0.22		

**Table 3:** Performance measures for mesothelin, miR-103a-3p, and their combination, calculated for maximum Youden's index (YI) and a false-positive rate (FPR) of 4%.

			Cut-off	N	Sensitivity	Specificity	True-positive	True-negative	False-positive	False-negative
					(%)	(%)	(N)	(N)	(N)	(N)
Mesothelin	All subjects	Maximum YI	1.70 nmol/l	95	60	92	26	48	4	17
		FPR = 4%	2.01 nmol/l	95	49	96	21	50	2	22
	Without sarcomatoid mesothelioma	Maximum YI	1.49 nmol/l	90	74	85	28	44	8	10
		FPR = 4%	2.01 nmol/l	90	55	96	21	50	2	17
miR-103a-3p	All subjects	Maximum YI	749.61	95	86	63	37	33	19	6
		FPR = 4%	99.73	95	7	96	3	50	2	40
	Without sarcomatoid mesothelioma	Maximum YI	749.61	90	89	63	34	33	19	4
		FPR = 4%	99.73	90	8	96	3	50	2	35
Combination of mesothelin and miR-103a-3p	All subjects	Maximum YI	−0.25≤−1.45–0.002 * 2^−dCt^+1.73 * mesothelin	95	86	85	37	44	8	6
		FPR = 4%	0.65≤−1.45–0.002 * 2^−dCt^+1.73 * mesothelin	95	65	96	28	50	2	15
	Without sarcomatoid mesothelioma	Maximum YI	−0.63≤−2.10–0.002 * 2^−dCt^+2.34 * mesothelin	90	95	81	36	42	10	2
		FPR = 4%	0.42≤−2.10–0.002 * 2^−dCt^+2.34 * mesothelin	90	74	96	28	50	2	10

Excluding sarcomatoid mesothelioma from analysis revealed a median mesothelin concentration of 2.03 nmol/l (IQR 1.24–5.82 nmol/l) and a median miR-103a-3p value of 228.5 (IQR 144.2–608.5) for mesothelioma patients. Differences between mesothelioma patients and asbestos-exposed controls were still statistically significant for both biomarkers (p<0.001).

### Mesothelin and miR-103a-3p as biomarkers for mesothelioma

**Figure 3:**
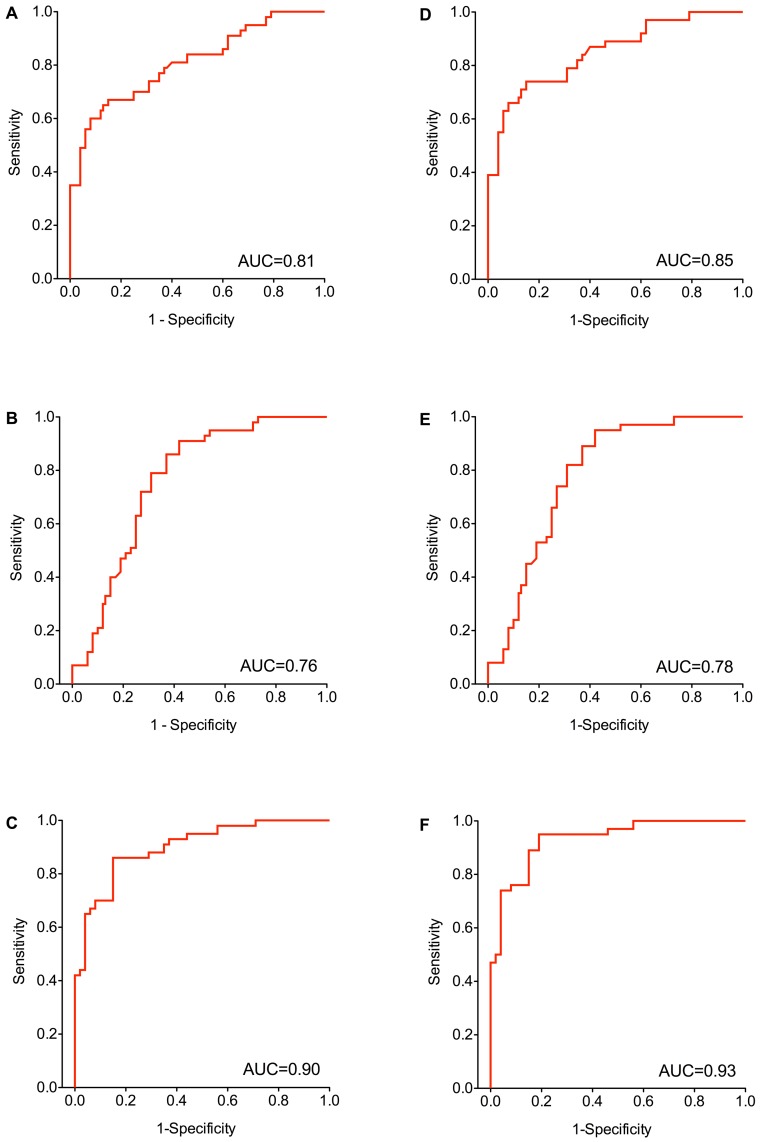
Receiver operating characteristics (ROC) curves of mesothelin and miR-103a-3p. The area under curve (AUC) was determined for all subjects (A–C) for mesothelin (A), miR-103a-3p (B), and the combination of mesothelin and miR-103a-3p (C), and for subjects without sarcomatoid mesothelioma (D–F) for mesothelin (D), miR-103a-3p (E), and the combination of mesothelin and miR-103a-3p (F).

Using ROC analyses including all subjects AUCs of 0.81 for mesothelin (Figure 3 A), 0.76 for miR-103a-3p (Figure 3 B), and 0.90 for the combination of mesothelin and miR-103a-3p (Figure 3 C) were calculated. Excluding the five sarcomatoid mesothelioma cases from the analysis revealed higher AUCs of 0.85 for mesothelin (Figure 3 D), 0.78 for miR-103a-3p (Figure 3 E), and 0.93 for the combination of mesothelin and miR-103a-3p (Figure 3 F).

Sensitivities and specificities for mesothelin and miR-103a-3p are shown in Table 3. Using maximum YI for cut-off selection resulted in 60% sensitivity and 92% specificity for mesothelin and 86% sensitivity and 63% specificity for miR-103a-3p. Excluding the five sarcomatoid mesothelioma cases resulted in 74% sensitivity and 85% specificity for mesothelin and 89% sensitivity and 63% specificity for miR-103a-3p. Additionally, cut-offs were calculated at a FPR of 4%, representing two false-positive tests. This resulted in lowered sensitivities, 49% for mesothelin and 7% for miR-103a-3p. Excluding the sarcomatoid mesothelioma cases resulted in sensitivities of 55% for mesothelin and 8% for miR-103a-3p. Notably, utilizing the recommended cut-off for mesothelin (1.5 nmol/l) [9] resulted in 65% sensitivity and 85% specificity for all subjects and 71% sensitivity and 85% specificity for subjects without sarcomatoid mesothelioma.

Combination of mesothelin and miR-103a-3p resulted in continuously higher sensitivities and consistently high specificities (Table 3). Using maximum YI resulted in 86% sensitivity and 85% specificity. Notably, four of five sarcomatoid mesothelioma cases were tested false-negative by the combination of the two biomarkers, but only one biphasic and one epithelioid mesothelioma. Excluding sarcomatoid mesothelioma from the analysis resulted in an increased sensitivity of 95% and specificity of 81%. Using a FPR of 4% resulted in 65% sensitivity for all subjects and 74% for subjects without sarcomatoid mesothelioma.

ROC analyses of 1,000 bootstrap samples resulted in similar sensitivities and specificities for the combination of mesothelin and miR-103a-3p in comparison to the original analyses (Table 4). The calculated 95% CIs indicated a good precision of this assessment.

**Table 4:** Estimates of ROC analyses with 95% CI for mesothelioma after bootstrap analysis with 1,000 random samples according to maximum Youden's index (YI) and a false-positive rate (FPR) of 4% for the combination of mesothelin and miR-103a-3p.

		N	Sensitivity (%)	95% CI	Specificity (%)	95% CI
All subjects	Maximum YI	95	84	64; 96	87	71; 98
	FPR = 4%	95	65	37; 83	96	92; 97
Without sarcomatoid mesothelioma	Maximum YI	90	92	74; 100	87	74; 98
	FPR = 4%	90	72	44; 90	96	91; 97

## Discussion

Mesothelioma is an aggressive cancer, commonly diagnosed at late stages of the disease. Reliable blood-based biomarkers could improve detection of mesothelioma at early stages [10]. The main limitation of an individual biomarker is the relatively low diagnostic performance, e.g., for mesothelin a sensitivity of 58%, a specificity of 89%, and an AUC of 0.85 were calculated in a meta-analysis [15]. A combination of two or more biomarkers within a panel might increase sensitivity and, if specificity remains sufficiently high, could thus improve the overall diagnostic performance.

In this study mesothelin was combined with miR-103a-3p, resulting in an improved AUC of 0.93. According to the maximum YI a sensitivity of 95% and a specificity of 81% was calculated. Based on a FPR of 4%, representing 96% specificity, the sensitivity was 74%. The obtained results are based on relatively small study groups but bootstrap analysis with 1,000 random samples showed that the calculated sensitivities and specificities remained stable. Thus, it might be reasonable to test the combination in larger study groups.

In recent years, several analyses were performed combining mesothelin with a number of different proteins. But none of the tested combinations showed a sufficient improvement of the biomarker performance. Whereas no advancement was shown for the combination of mesothelin with CA125 [19] or CA125 and CYFRA21-1 [20], improvements were revealed for the combinations of mesothelin with YKL-40 (72% Sensitivity, 84% Specificity, AUC = 0.86) [21], osteopontin (85% sensitivity, 90% specificity, AUC = 0.87) [22], and CEA (56% sensitivity, 95% specificity, AUC = 0.89) [23]. These relatively moderate improvements already indicate that it is likely that biomarkers of the same molecular class mostly discriminate the same subjects. In contrast, Santarelli et al. indicated that the combination of mesothelin with a different molecular class, namely miRNAs, improved the diagnostic performance more distinctly [12] as this is also shown in this study.

The combination of mesothelin and miR-103a-3p appears to be more complementary than a combination of biomarkers within the same molecular class. This might be due to differences between the mechanisms how biomarkers are released into blood. Generally, proteins are released during natural secretion processes, which can be altered by cancer. In contrast, DNA and RNA are mostly released during apoptosis, necrosis, and other processes. Therefore, a complementary effect between biomarkers of different molecular classes should be more likely and might result in an enhanced discrimination between cases and controls. Also, miR-103a-3p was not isolated from plasma but from the cellular fraction of blood. Thus, it has to be considered that changes in levels of miR-103a-3p may be the result of an indirect effect, i.e., a response of the immune system to the tumor.

For mesothelin a role in cell adherence, proliferation, and cancer progression has been implied [24] but less is known about miR-103a-3p. Recently, miR-103a-3p was shown to control the expression of *GPRC5A* mRNA and its protein product in pancreatic cells [25]. *GPRC5A* acts as oncogene or tumor suppressor in different types of cancer [26] but nothing is known about *GPRC5A* in mesothelioma. Thus, it might be reasonable to evaluate the potential roles of miR-103a-3p and *GPRC5A* in mesothelioma.

Using immunohistochemistry, breast carcinoma, particularly triple-negative breast cancer that metastasize to the pleura and lung, might be confused with mesothelioma [27]. Recently, it was shown that in tissues of patients with triple-negative breast cancer and distant metastases the miR-103a-3p expression is slightly elevated [28]. In contrast, miR-103a-3p is *down*regulated in the blood of mesothelioma patients [14]. Thus, it might be interesting to evaluate whether miR-103a-3p is also a possible candidate for the differential diagnosis of mesothelioma and pleural metastasis of breast cancer.

The use of individual biomarkers for diagnosis is frequently characterized by relatively low sensitivities and/or specificities. This is also true for miR-103a-3p and mesothelin alone. Generally, high specificities are required in cancer screening [8]. Low specificities might increase sensitivities but result in higher rates of false-positive tests in non-diseased subjects. This can lead to psychological pressure and unnecessary diagnostic interventions, which should be avoided in screening programs. However, an acceptable FPR also depends on the fatality of the disease and the invasiveness of the diagnostic procedure [8]. The FPR of 4% used in this study resulted in two false-positive tests. One subject had a biliary colic and a stroke, whereas the second subject had a thyroid dysfunction. Indeed, miR-103 was shown to be involved in thyroid carcinoma [29] and stroke [30], which could possibly be influencing factors that caused the false-positive tests. However, the impact of biliary colic, stroke, and thyroid dysfunction on biomarker levels needs to be verified in detail. Still, as the examined controls were formerly exposed to asbestos it cannot be ruled out that these two subjects might develop a malignant disease in the future.

It is well known that mesothelin fails to detect sarcomatoid mesothelioma [9], [31] and this is also shown in this study. In particular, a high OR of 72 was calculated for sarcomatoid mesothelioma cases to be tested false-negative. Sarcomatoid mesothelioma is not the predominantly histological subtype, counting for only 10–20% of all cases whereas the epithelioid and biphasic subtypes comprised 50–60% and 25–35%, respectively [32]. Thus, we focused on the most common subtypes, epithelioid and biphasic mesothelioma.

The knowledge of biological, pre-analytical, and analytical factors influencing the biomarker levels is important for the assessment of the reliability of a biomarker [33]. In recent years, circulating miRNAs in plasma and serum have shown potential as valuable biomarkers for the detection of several cancers. However, Kirschner et al. reported that a number of the proposed miRNAs are influenced by hemolysis rather than cancer [34]. Even if plasma samples are not visible colored, hemolysis might be present and can greatly increase the levels of certain miRNAs. In order to avoid misleading results, it is crucial that each miRNA is tested prior to analysis whether it is affected by hemolysis. However, miR-103a-3p is not influenced by hemolysis as shown recently [34]. As a possible influencing factor for mesothelin, Beyer et al. detected higher mesothelin levels in subjects with hypertension [9]. This is in contrast to our study where lower concentrations of mesothelin were observed in patients with hypertension. The difference was probably caused by different study designs, mostly regarding the size and composition of the study groups. However, it cannot be ruled out that hypertension has a relevant impact on mesothelin levels. The OR for hypertension cases to be tested false-negative was 2 (95% CI 0; 9) but was not significant (data not shown). However, six out of ten mesothelioma cases that tested false-negative in this study had hypertension. For a more detailed evaluation of the impact of potential influencing factors the multiplicity of biological, pre-analytical, and analytical factors have to be included. Such an analysis has to be performed in a sufficiently large study group of healthy subjects without malignant diseases [8], [35].

For translational medicine it is essential that potential biomarkers are measurable in routine clinical practice. For mesothelin it was shown that the used ELISA is applicable for the measurement on automated systems [36] and for miRNAs it has been suggested that appropriate miRNAs might be implemented in FDA-approved kit-based assays in the near future [37].

In conclusion, we showed that the combination of mesothelin and miR-103a-3p improved the diagnostic performance of a blood-based screening test, resulting in higher sensitivity and specificity to detect malignant mesothelioma. The results support the concept that combinations of rather than individual biomarkers are needed for a reliable diagnosis of malignant diseases. In addition, it seems especially promising to use combinations of markers from different molecular classes, i.e. proteins and nucleic acids, to improve diagnostic performance.

## Supporting Information

Table S1 **Subjects characteristics, raw data of miR-103a-3p and miR-125a expression analysis, and mesothelin concentration.**
(XLSX)
